# The Nurse's Library

**Published:** 1921-02-26

**Authors:** 


### THE NURSE'S LIBRARY.

**A Nurse's Handbook of Obstetrics.** By J. B. Cooke, M.D. Ninth Edition, revised by C. E. Gray, R.N., and P. F. Williams, M.D. (Lippincott, 1920. Pp. 468. Price 12s. 6d. net.)

A text-book that has reached nine editions, especially in a country less conservative than Britain is, more impatient of authority, and more avid of change for the sake of change (as distinct from progress), must possess definite and conspicuous merits. Examination of this book shows what they are: dogmatic teaching, essentially of a practical character, and expressed in terms the most readily comprehensible by those for whom it is intended; thoroughness in dealing with the fundamental principles of the subject, without wasting too much time in the explanation of side issues; and a high standard of professional conduct constantly held up as the ideal for the novice to aspire towards. Dr. Cooke and his two editors waste no time over non-essentials: they "get to the 'osses" with true American decision and promptitude. It does seem, however, as if some of the necessary revision of this long-established text-book has been not quite sufficiently drastic: there are a few passages which seem to reflect the teaching of a past generation rather than of the present day. Two-hourly feeds are advocated for infants from birth up to six weeks of age, instead of the three-hour intervals now in vogue (and rightly for the majority of babies). The pregnant woman is advised to send to her physician for examination eight ounces of sterile urine from a twenty-four-hour collection, a piece of advice with which it is absolutely and entirely impossible for her to comply. It is stated that noxious fumes will not affect the larynx of an anæsthetised woman, although they may seriously affect the larynxes of her attendants—a patently absurd statement. It is asserted, without the least justification or probability so far as we are aware, that rickets in childhood is due to too much fruit in the diet of the pregnant mother. Labour is described as being always protracted when the membranes rupture early. And this list of solecisms could be extended. Let us turn, however, to a feature for which unqualified approval can be expressed—namely, the photographs in colour representing normal and abnormal stools of nurslings. Here is an idea which we should be glad to see copied in British text-books.

**A Short History of Nursing: from the Earliest Times to the Present Day.** By Lavinia L. Dock, R.N., in collaboration with Isabel Maitland Stewart, A.M., R.N. (New York and London: G. P. Putnam's Sons. 1920. Price 17s. 6d.)

This volume is a condensation of the larger "History of Nursing" in four volumes, prepared by Miss Dock and Miss Nutting, and has greatly benefited by the excision and compression exercised. The earlier chapters on the "Care of the Sick in the Ancient World," "Christianity and the Care of the Sick," "The Military Influences," "Democratic and Secular Tendencies," "The Dark Period in Nursing," and "Florence Nightingale" are by far the most interesting. English readers will find much that is new to them in the story of the rise and spread of trained nursing in America, and the extensions of the modern nursing field on that continent, with its recent educational developments. The account of nursing in other countries, including Great Britain, is exceedingly meagre, and the brief reference to the College of Nursing cannot but provoke a smile. An excellent chapter on "Past and Future" emphasises the extent to which military etiquette has been stamped on the nursing service, and claims it as "one of our proudest traditions that nurses have raised so much of what used to be called menial work to the rank of a science and art."

**The Care of Children: A Guide for Mothers and Nurses at Home and Abroad.** By Robert J. Blackham, C.B., C.M.G., C.I.E., D.S.O., M.D. (Scientific Press, Ltd., 28 and 29 Southampton Street, Strand. Price 4s. 6d. net.)

The fourth edition of this admirable book has just been announced, revised, enlarged, and largely rewritten, and such of our readers as are in need of a safe handbook to put into the hands of young mothers can find nothing better suited to their purpose. The problems of childhood are discussed in a manner both wise and interesting. A great number of popular fallacies, harmful to infants yet commonly acted upon with the best intentions, are adroitly demolished, and among them that of drugging. Colonel Blackham is too experienced in the ways of fond mothers to be sanguine about restraining them altogether from administering medicines to their offsprings, but he has given them a choice of fifteen harmless concoctions, beginning with dill water, beyond which they are not to stray. The hygiene is clearly explained and eminently practical. One of the special features of the book is its hints on the management of children in hot climates, a branch of instruction for which there is great need.

**The Theory and Practice of Massage.** By Beatrice M. Goodall-Copestake. Third Edition. With 69 illustrations, including 20 plates. (London: H. K. Lewis & Co., Ltd. 1920.)

A chapter on the re-education of muscles, an important branch of remedial gymnastics, has been added to this edition, for which the author expresses indebtedness to Miss Ryde. Some diagrams, prepared by Mr. Gilbert Scott, of the position of the stomach and colon in health and disease are an interesting new feature, as are also the $x$-ray plates lent by Mr. Wilding. Miss Copestake's accuracy and lucid mode of imparting instruction are too well known to countless students and teachers to require commendation. Her book continues to be one of the standard works in preparation for examinations, and as an aid to memory in subsequent practice.

**Black's Simple Cookery and Household Management.** Edited by the Edinburgh School of Cookery and Domestic Economy. (A & C. Black, Ltd., 4, 5, and 6 Soho Square, W. 1. 1920. Price 5s.)

During the war the Edinburgh School of Cookery did excellent service in issuing leaflets on economy, with recipes for vegetable and cereal dishes which sold by hundreds of thousands. These leaflets were afterwards collected, and proved very popular under the name of "Savings and Savoury Dishes." They have now been carefully edited, the war-time aspect has been eliminated, and the result is a valuable contribution to the art of living well at a moderate cost. For all who have to bring up a family or provide for a large household at a minimum cost the recipes and advice contained in this manual will be found of substantial service. By using meat as an adjunct to a meal rather than as its main ingredient, and by suggesting a great variety of appetising dishes containing no meat at all, the editors widen the range of available foodstuffs, and if their advice were intelligently followed it would reduce the cost of living certainly by one-third, possibly by half. We particularly commend the dietary for little children.

